# Comparison of ePlex Respiratory Pathogen Panel with Laboratory-Developed Real-Time PCR Assays for Detection of Respiratory Pathogens

**DOI:** 10.1128/JCM.00221-17

**Published:** 2017-05-23

**Authors:** R. H. T. Nijhuis, D. Guerendiain, E. C. J. Claas, K. E. Templeton

**Affiliations:** a Department of Medical Microbiology, Leiden University Medical Center, Leiden, The Netherlands; b Specialist Virology Center, Royal Infirmary of Edinburgh, Edinburgh, United Kingdom; Boston Children's Hospital

**Keywords:** influenza, point-of-care testing, rapid diagnostics, respiratory pathogens, sample-to-answer

## Abstract

Infections of the respiratory tract can be caused by a diversity of pathogens, both viral and bacterial. Rapid microbiological diagnosis ensures appropriate antimicrobial therapy as well as effective implementation of isolation precautions. The ePlex respiratory pathogen panel (RP panel) is a novel molecular biology-based assay, developed by GenMark Diagnostics, Inc. (Carlsbad, CA), to be performed within a single cartridge for the diagnosis of 25 respiratory pathogens (viral and bacterial). The objective of this study was to compare the performance of the RP panel with those of laboratory-developed real-time PCR assays, using a variety of previously collected clinical respiratory specimens. A total of 343 clinical specimens as well as 29 external quality assessment (EQA) specimens and 2 different Middle East respiratory syndrome coronavirus isolates have been assessed in this study. The RP panel showed an agreement of 97.4% with the real-time PCR assay regarding 464 pathogens found in the clinical specimens. All pathogens present in clinical samples and EQA samples with a threshold cycle (*C_T_*) value of <30 were detected correctly using the RP panel. The RP panel detected 17 additional pathogens, 7 of which could be confirmed by discrepant testing. In conclusion, this study shows excellent performance of the RP panel in comparison to real-time PCR assays for the detection of respiratory pathogens. The ePlex system provided a large amount of useful diagnostic data within a short time frame, with minimal hands-on time, and can therefore potentially be used for rapid diagnostic sample-to-answer testing, in either a laboratory or a decentralized setting.

## INTRODUCTION

Infections of the upper and lower respiratory tract can be caused by a diversity of pathogens, both viral and bacterial. Community-acquired respiratory tract infections are a leading cause of hospitalization and responsible for substantial morbidity and mortality, especially in infants, the elderly, and immunocompromised patients. The etiological agent in such infections differs greatly according to season and age of patient, with highest prevalences being those of respiratory syncytial virus (RSV) in children and influenza virus in adults. Rapid microbiological diagnosis of a respiratory infection is important to ensure appropriate antimicrobial therapy and for the effective implementation of isolation precautions (1).

In the last decade, many conventional diagnostic methods such as culture and antigen detection assays have been replaced by molecular assays for diagnosing respiratory tract infections. Multiplex real-time PCR assays have been developed and implemented for routine diagnostic application, detecting a wide variety of pathogens (2–7). These assays have shown high sensitivity and specificity, but the limited number of fluorophores that can be used per reaction resulted in the need to run several real-time PCR assays to cover a broad range of relevant pathogens. Commercial assays using multiplex ligation-dependent probe amplification (MLPA), a dual priming oligonucleotide system (DPO), or a microarray technology were developed to overcome this problem and are able to detect up to 19 viruses simultaneously (8, 9). All applications mentioned require nucleic acid extraction prior to amplification. For routine diagnostics, these methods are most suited for batch-wise testing, with a turnaround time of ∼6 to 8 h. To decrease the time to result and enable random access testing, syndromic diagnostic assays have been developed. These assays combine nucleic acid extraction, amplification, and detection in a single cartridge per sample and are suitable for decentralized or even point-of-care testing (POCT) with a time to result of <2 h.

**FIG 1:**
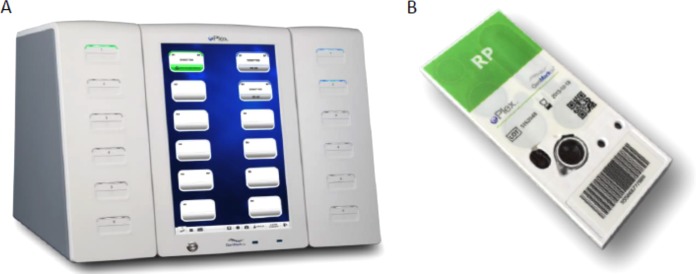
ePlex system (A) and the corresponding cartridge of the ePlex respiratory pathogen panel (B).

**FIG 2:**
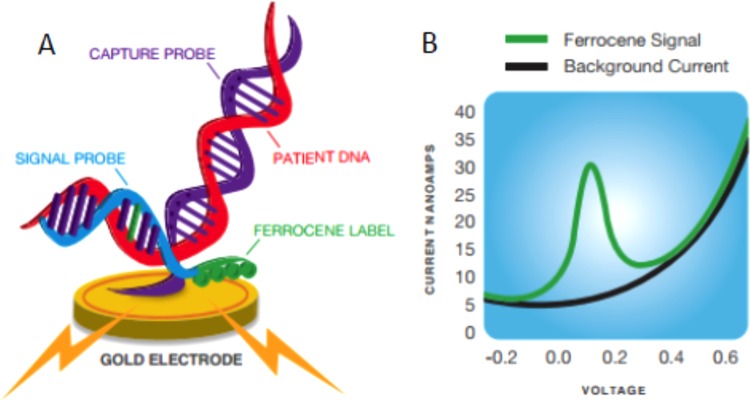
Principle of the eSensor detection technology. Amplified sequences of the targeted pathogens are detected electrochemically using a complementary pathogen-specific signal probe tagged with ferrocene, a reducing agent. The hybridized molecule is then exposed to another sequence-specific probe that is bound to a solid phase, which is a gold electrode (A). Upon binding of the two molecules, the ferrocene comes in close proximity to the gold electrode, where an electron transfer that can be measured using GenMark's eSensor technology on the ePlex system can occur (B).

A novel rapid diagnostic, cartridge-based assay for the detection of respiratory tract pathogens using the ePlex system (Fig. 1) was developed by GenMark Diagnostics, Inc. (Carlsbad, CA). The ePlex respiratory pathogen panel (RP panel) is based on electrowetting technology, a digital microfluidic technology by which droplets of sample and reagents can be moved efficiently within a network of contiguous electrodes in the ePlex cartridge, enabling rapid thermal cycling for a short time to result. Following nucleic acid extraction and amplification, detection and identification are performed using the eSensor detection technology (Fig. 2), as previously applied in the XT-8 system (10).

In the current study, the performance of the syndromic RP panel was compared to those of laboratory-developed real-time PCR assays, using clinical specimens previously submitted for diagnosis of respiratory pathogens.

## RESULTS

**FIG 3:**
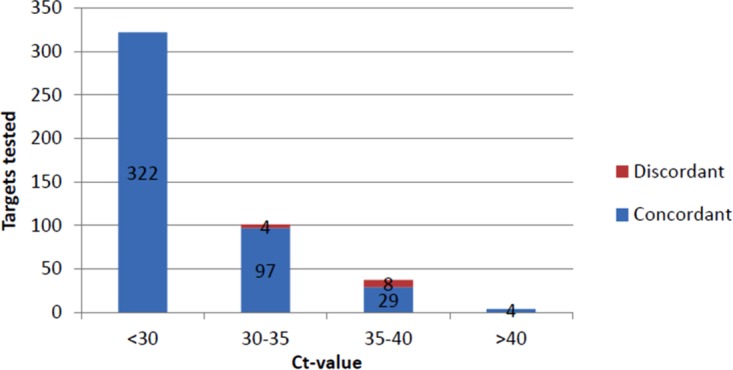
Pathogen concordance by *C_T_* value.

The 323 positive clinical specimens contained a total of 464 respiratory pathogens as detected by laboratory-developed real-time PCR assays (Table 1). As shown in Table 2, the 57 nonnasopharyngeal (non-NPS) specimens comprised 69 of the total 464 respiratory pathogens. Testing all samples with the RP panel resulted in an overall agreement for 452 (97.4%) targets from 311 specimens, prior to discrepant analysis. Of the specimens containing a single pathogen, the detected targets were concordant in 209/217 specimens. For samples with coinfection, the same pathogens could be identified in 77/81, 22/22, and 3/3 in the case of 2, 3, and 4 pathogens present, respectively. Eight of 12 discordant targets (PCR^+^/RP^−^) had a positive result with threshold cycle (*C_T_*) values of >35 (Fig. 3). Retesting with a third assay confirmed 10 of 12 real-time PCR-positive targets being human bocavirus (hBoV; *n* = 3), rhinovirus (RV; *n* = 2), parainfluenza virus type 2 (PIV2; *n* = 1)), human coronavirus (hCoV) OC43 (*n* = 1), hCoV 229E (*n* = 1), hCoV HKU1 (*n* = 1), and human metapneumovirus (hMPV; *n* = 1). The two unresolved PCR^+^/RP^−^ results consisted of two hMPV-positive samples (*C_T_* values of 33.2 and 38.3).

**TABLE 1:** Pathogens included in this study

RP panel target	No. of results		
	Found in clinical specimens	PCR^+^/RP^−^	PCR^−^/RP^+^
Viral			
Adenovirus	39		4
Coronavirus			
229E	7	1	1
HKU1	12	1	
NL63	7		1
OC43	9	1	1
MERS coronavirus			
Human bocavirus	27	3	1
Human metapneumovirus	28	3	
Human rhinovirus/enterovirus^*a*^	134^*b*^	2^*c*^	6^*a*^
Influenza virus			
Influenza A	1^*d*^		
H1	5		
2009 H1N1	18		1^*e*^
H3	17		
Influenza B	20		
Parainfluenza virus			
Type 1	11		
Type 2	12	1	
Type 3	15		
Type 4	2		2
Respiratory syncytial virus	13^*d*^		
Type A	43		
Type B	24		
Bacterial			
Bordetella pertussis	6		
Chlamydophila pneumoniae	0		
Legionella pneumophila	6		
Mycoplasma pneumoniae	8		

a No differentiation possible between rhinovirus and enterovirus.

b One hundred seven rhinoviruses and 27 enteroviruses found by routine testing.

c Both rhinovirus.

d No further subtyping performed.

e In the laboratory-developed test detected as influenza A virus.

**TABLE 2:** Non-NPS^*a*^ specimens included in this study

Specimen type	No. included	Pathogen (*n*)
Sputum	21	Human rhinovirus (10)
		Legionella pneumophila (6)
		Human metapneumovirus (3)
		Respiratory syncytial virus (1)
		Influenza A virus H1N1 (1)
		Influenza A virus H3 (1)
		Mycoplasma pneumoniae (1)
Bronchoalveolar lavage fluids	16	Human rhinovirus (12)
		Human metapneumovirus (3)
		Enterovirus (2)
		Human bocavirus (2)
		Respiratory syncytial virus (1)
		Mycoplasma pneumoniae (1)
		Adenovirus (1)
Throat swab	10	Human rhinovirus (7)
		Respiratory syncytial virus (4)
		Enterovirus (1)
Nasopharyngeal aspirate	10	Respiratory syncytial virus (7)
		Human rhinovirus (3)
		Enterovirus (1)
		Adenovirus (1)

a NPS, nasopharyngeal swab.

The RP panel yielded a positive result in 17 specimens, where the laboratory-developed test (LDT) remained negative (PCR^−^/RP^+^), including 15 additional pathogens previously undetected by LDT in the 323 positive specimens and one influenza A H1N1 2009 virus that was detected as influenza A virus by LDT (Table 1). Seven of these 15 additional targets could be confirmed, including three of RV/enterovirus (EV) (all confirmed as RV), two of PIV4, and one each of hBoV and hCoV NL63.

One of the selected negative samples tested positive for human adenovirus (hAdV) in the RP panel but could not be confirmed by discrepant testing. All other negative specimens tested negative in the RP panel as well.

Both Middle East respiratory syndrome coronavirus (MERS-CoV) isolates could be detected by the RP panel. By testing a 10-fold dilution series of both isolates, it was shown that MERS-CoV with a *C_T_* value of <30 in the laboratory-developed real-time PCR assay could be detected using the RP panel, while detection with a *C_T_* value of >30 was achievable but was not reproducible in every instance.

Of the 12 specimens from the Quality Control for Molecular Diagnostics (QCMD) 2016 Respiratory II Pilot external quality assessment (EQA) study panel, 10 were detected in full agreement with the content as reported by QCMD (Table 3). The 2 false-negative tested specimens both contained hCoV NL63, of which one was a coinfection in an hMPV-positive sample. Both specimens had been tested with the laboratory-developed real-time PCR assay as well and were found positive for hCoV NL63, both with *C_T_* values of 37.4.

**TABLE 3:** EQA samples included in the study

EQA sample	Content^*a*^	RP panel
RESPII16-01	Rhinovirus type 72 d1	Rhinovirus/enterovirus
RESPII16-02	Rhinovirus type 72 d2	Rhinovirus/enterovirus
RESPII16-03	Negative	Negative
RESPII16-04	Adenovirus type 1	Adenovirus
RESPII16-05	Coronavirus OC43	Coronavirus OC43
**RESPII16-06**^*b*^	**Human metapneumovirus and coronavirus NL63**	**Human metapneumovirus**
RESPII16-07	Rhinovirus type 8	Rhinovirus/enterovirus
RESPII16-08	Parainfluenza virus type 3 and rhinovirus type 72	Parainfluenza virus type 3 and rhinovirus/enterovirus
RESPII16-09	Human metapneumovirus	Human metapneumovirus
RESPII16-10	Coronavirus NL63 d1	Coronavirus NL63
**RESPII16-11**	**Coronavirus NL63 d2**	**Negative**
RESPII16-12	Parainfluenza virus type 1	Parainfluenza virus type 1
Qnostics RSP-S01	Influenza A virus	Influenza A virus
Qnostics RSP-S02	Influenza B virus	Influenza B virus
Qnostics RSP-S03	Respiratory syncytial virus type A	Respiratory syncytial virus type A
Qnostics RSP-S04	Respiratory syncytial virus type B	Respiratory syncytial virus type B
**Qnostics RSP-S05**	**Adenovirus type 1**	**Negative**
Qnostics RSP-S06	Coronavirus NL63	Coronavirus NL63
Qnostics RSP-S07	Coronavirus OC43	Coronavirus OC43
Qnostics RSP-S08	Parainfluenza virus type 1	Parainfluenza virus type 1
Qnostics RSP-S09	Parainfluenza virus type 3 and rhinovirus	Parainfluenza virus type 3 and rhinovirus/enterovirus
Qnostics RSP-S10	Human metapneumovirus	Human metapneumovirus
Qnostics RSP-S11	Rhinovirus	Rhinovirus/enterovirus
Qnostics RSP-S12	Bordetella pertussis	Bordetella pertussis
**Qnostics RSP-S13**	**Chlamydophila pneumoniae**	**Negative**
Qnostics RSP-S14	Mycoplasma pneumoniae	Mycoplasma pneumoniae
Qnostics RSP-S15	Legionella pneumophila	Legionella pneumophila
Qnostics RSP-S16	Legionella pneumophila	Legionella pneumophila
Qnostics RSP-S17	Negative	Negative

a d1 and d2, different dilutions of the same virus, no information on the concentration provided.

b Data in bold indicate samples that incorrectly detected the (full) content of the EQA sample.

The Qnostics evaluation panel consisted of 17 samples, including 15 different respiratory pathogens and one negative sample (Table 3). The RP panel detected 15 of the specimens in agreement with the content, whereas hAdV type 1 and Chlamydophila pneumoniae were not detected. Real-time PCR detection of these specimens was performed to confirm the presence of the respective pathogen in the specimen and was found positive for both hAdV (*C_T_* value of 31.4) and C. pneumoniae (*C_T_* value of 35.4).

## DISCUSSION

The performance of the ePlex RP panel was assessed by retrospective testing of 343 clinical respiratory specimens (obtained in 2009 to 2016) comprising five different types of specimens. Although the RP panel had been CE *in vitro* diagnostic (CE-IVD) cleared for detection of respiratory pathogens from NPS swabs only, we included a range of alternate sample types that can be obtained and tested for respiratory pathogens in the diagnostic setting. By including a total of 57 respiratory non-NPS specimens with different pathogens (Table 2), it was shown that the RP panel was able to accurately detect the pathogen(s) in the different types of specimens, as the assay showed 100% concordance with LDT. For sputum samples, preprocessing with Sputasol was introduced after the initial 6 tested specimens, since 1 false-negative result was found, which was resolved on retesting with Sputasol pretreatment. Further studies need to determine the frequency of preprocessing of sputum samples before efficiently running the RP panel.

Specimens for inclusion in this study were previously tested at two different sites, using both their own systems and validated assays. Although the initial setups of the LDT assays were the same (11, 12), minor adjustments of the assays and the use of different PCR platforms may affect the performance of the LDTs and therefore were a limitation of this study.

Comparison of the results from the RP panel with the results from the routine multiplex real-time PCR showed an agreement of 97.4% in 464 pathogens tested. Figure 3 shows that PCR^+^/RP^−^ targets are mainly targets with a low viral or bacterial load (based on *C_T_* values). Analysis of real-time PCR results with *C_T_* values of <35 provided an agreement between the RP panel and laboratory-developed real-time PCR of 99.1%. Specimens containing pathogens with a high load (*C_T_* value of <30) were all detected correctly by the RP panel, independently of the type of specimen, type of pathogen, or the number of different pathogens in a specimen. This finding is in line with earlier evaluation of the GenMark XT-8 system using the same eSensor principle of detection (10). With 29 concordant targets with a *C_T_* value between 35 to 40 and 4 targets with a *C_T_* value of >40, the RP panel showed good detection rates with regard to lower viral or bacterial loads as well (Fig. 3).

Although the performance of the RP panel appeared to be excellent using the tested specimens in this study, for PIV4 (*n* = 2) and C. pneumoniae (*n* = 0) the number of clinical specimens that could be analyzed was too low for a proper assessment of the assay, which was a limitation of this study.

In 14 different specimens, the RP panel identified 15 pathogens that had not been detected by routine testing (PCR^−^/RP^+^). In addition, one influenza A virus detected by LDT could be detected as influenza A H1N1 2009 virus by the RP panel. One of the selected negative samples was shown to contain an hAdV, while all other PCR^−^/RP^+^ targets were detected as copathogens to other positive targets in the samples. All the PCR^−^/RP^+^ targets were found in samples obtained from 1 institute. Discrepant analysis on the eluates of these samples was performed using the LDT of the other institute, where seven of the additionally detected pathogens could be confirmed by discrepant testing. These samples showed a relatively low viral load based on the mean *C_T_* value found (33.3), probably around the limit of detection of the initial LDT. It was unclear whether the 8 unresolved PCR^−^/RP^+^ targets are false positive or the results of more efficient detection of multipathogen infections by the eSensor technology (10).

A small number of LDT-negative specimens (*n* = 20) was included in this study since the main objective of this study was to determine the performance of the RP panel in detecting respiratory pathogens. Although this is a limitation of the current study, we believe that this issue will be addressed extensively in upcoming prospective clinical studies.

Owing to the lack of clinical specimens containing MERS-CoV, dilutions of two different culture isolates were tested in this study, of which dilutions with *C_T_* values of <30 as shown by the laboratory-developed real-time PCR assay could be detected consistently. It should be noted that the real-time PCR assay has been developed for research use and has not yet been validated for clinical use.

Assessment of the RP panel using EQA samples from QCMD and Qnostics showed results that are in line with the results obtained from clinical specimens. A total of 4 targets included in the EQA samples could not be detected using the RP panel, showing *C_T_* values of >35 (*n* = 3) and 31.4 (*n* = 1) when tested by real-time PCR.

The RP panel on the ePlex system enables rapid testing and can be used as a diagnostic system in either a laboratory or a decentralized setting that is closer to the patient. The assay turned out to be rapid and straightforward to perform. Compared to routine testing, hands-on time of the RP panel was very low (<2 min), whereas the hands-on time of the routine testing was about 30 to 45 min, depending on the nature and number of samples tested. The overall run time of the platforms was also in favor of the ePlex system, as it takes approximately 90 min for nucleic acid extraction, amplification, hybridization, and detection, whereas routine testing takes up to 2 h and 45 min using different systems and multiple real-time PCR assays in multiplex. An important advantage of the ePlex system is the possibility of random access testing, compared to batch-wise testing in the current diagnostic real-time PCR approach. With a relatively short turnaround time and the potential to randomly load and run up to 24 specimens, the ePlex system is very suitable for testing STAT samples, which require immediate testing. In contrast to LDTs, where *C_T_* values represent a quantitative indicator, the ePlex system generates qualitative results only. The *C_T_* value is dependent on many different factors such as sample type and course of infection and can therefore differ greatly, even within a single patient. Hence, a qualitative result, e.g., identification of the pathogen, is the major factor for patient management.

The costs of reagents per sample are relatively high for ePlex compared to LDT. However, when taking into account the hands-on time of technicians and the clinical benefit of more rapid results, the assay will most likely be more cost-effective. Studies evaluating a rapid diagnostic assay for respiratory pathogens, such as the FilmArray respiratory panel (BioFire Diagnostics, Salt Lake City, UT), have already shown the impact of rapid diagnostics for respiratory pathogens, since it decreased the duration of antibiotic use, the length of hospitalization, and the time of isolation, delivering financial savings (13, 14). Although the RP panel on the ePlex system has the same potential, clinical studies remain to be conducted to fulfill this potential.

In conclusion, this study shows excellent performance of the GenMark ePlex RP panel in comparison to laboratory-developed real-time PCR assays for the detection of respiratory pathogens from multiple types of clinical specimens and EQA samples. The system provides a large amount of useful diagnostic data within a short time frame, with minimal hands-on time, helping to reduce laboratory costs for labor and deliver a faster result to the clinician in order to aid in appropriate antimicrobial therapy. Therefore, this syndrome-based diagnostic assay could be used as rapid diagnostic testing in many different settings.

## MATERIALS AND METHODS

### Specimens.

Clinical specimens selected for this study have previously been submitted and tested prospectively for diagnosis of respiratory infections at either the Specialist Virology Center at the Royal Infirmary of Edinburgh (RIE) or the medical microbiology laboratory at the Leiden University Medical Center (LUMC). Specimens were selected using the laboratory information management system of the corresponding institute, without prior selection based on *C_T_* value. Ethical approval for this study was granted by the medical ethical committee provided that anonymized samples were used. A total of 343 clinical specimens (286 nasopharyngeal [NPS] swabs, 21 sputum samples, 16 bronchoalveolar lavage [BAL] fluid samples, 10 throat swabs, and 10 nasopharyngeal aspirates) were used for this study, 323 positive and 20 negative. In the absence of clinical samples, Middle East respiratory syndrome coronavirus (MERS-CoV) isolate Jordan/N3 and recombinant MERS-CoV isolate EMC/2012 were tested. Finally, the QCMD 2016 Respiratory II Pilot study panel (12 samples) and a custom external quality assessment (EQA) evaluation panel of 17 samples provided by Qnostics Ltd. (Glasgow, United Kingdom) were tested.

### Diagnostic testing by lab-developed tests.

In short, the routine testing method consisted of total nucleic acid extraction by the NucliSENS easyMAG system (∼45 min; bioMérieux, Basingstoke, United Kingdom) or the MagNA Pure LC system (∼45 min to 1 h 30 min depending on the number of samples; Roche Diagnostics, Almere, The Netherlands), at the RIE and the LUMC, respectively. An input volume of 200 μl per specimen and elution volume of 100 μl were used for all specimen types. Amplification and detection were performed by real-time PCR using the ABI 7500 fast thermocycler (1 h; Applied Biosystems, Paisley, United Kingdom) or the Bio-Rad CFX96 thermocycler (∼1 h 40 min; Bio-Rad, Veenendaal, The Netherlands), at the RIE and the LUMC, respectively. Real-time PCR assays were tested with updated versions (where needed) of primers and probes as described previously (11, 12).

### RP panel.

Original clinical specimens were retrieved from storage at −70°C and thawed at room temperature. After vortexing, 200 μl of the specimen was pipetted into the sample delivery device with a buffer provided by the manufacturer. For 16 out of 21 sputum samples, preprocessing was done using Sputasol (Oxoid, Basingstoke, United Kingdom) according to the manufacturer's procedures (with the exception of washing the sputum) and incubation at 37°C for 15 min on a shaker at 500 rpm. After gentle mixing of the specimen and buffer in the sample delivery device, the mixture was dispensed into the cartridge using the sample delivery port, which was subsequently closed by sealing with a cap. After scanning of the barcode of the ePlex RP panel cartridge and the barcode of the corresponding sample, the cartridge was inserted into an available bay of the ePlex system. The test then started automatically and ran for approximately 90 min.

A single cartridge of the RP panel is able to detect 25 respiratory pathogens, including differentiation of subtypes of influenza A virus, parainfluenza virus, and respiratory syncytial virus (RSV) (Table 1). Internal controls for extraction, bead delivery, and movement within the cartridge are present, as well as those for amplification, digestion, and hybridization of DNA and RNA targets. For every specimen tested, a sample detection report was created, comprising the results for all targets and internal controls. Results of the targets are reported as positive or not detected. If an internal control fails, this will be noted on the detection report and samples should be retested with a new cartridge.

### Discrepant testing.

In the case of discrepant results, the discordant sample was retested either with a new ePlex cartridge if the real-time PCR was positive and the RP panel was negative (PCR^+^/RP^−^) or with the laboratory-developed real-time PCR assay in the case of PCR^−^/RP^+^ results. For unresolved discrepancies, additional testing with a third PCR assay (different primers and probe) was performed for final resolution.
